# Prevalence of metronidazole resistance and *Helicobacter pylori* infection in Moroccan children: a cross-sectional study

**DOI:** 10.11604/pamj.2024.48.89.43271

**Published:** 2024-07-04

**Authors:** Fatima Zahra Kheir, Aicha Baalala, Ghizlane Bounder, Abdelhak Abkari, Dalal Ben Sabbahia, Meriem Atrassi, Halima Rchid, Nourdin Harich, Mariama Lasky, Hasna Boura

**Affiliations:** 1 Laboratory of *Helicobacter pylori* and Gastric Pathologies, Institut Pasteur du Maroc, Casablanca 20360, Morocco,; 2 Laboratory of Biotechnology and Valorization of Plant Resources, Faculty of Sciences, Chouaib Doukkali University, El Jadida 24000, Morocco,; 3 Laboratory of Anthropogenetics, Biotechnology and Health, Faculty of Sciences, University Chouaib Doukkali, El Jadida, 24000, Morocco,; 4 The Department of Pediatrics III, Unit of Gastroenterology and Hepatology Pediatric, Abderrahim Harrouchi, Children Hospital, Ibn Rochd University Hospital, Casablanca, Morocco

**Keywords:** *Helicobacter pylori*, prevalence, resistance, metronidazole, children

## Abstract

**Introduction:**

the prevalence of Helicobacter pylori (H. pylori) infection in children is very high in Morocco. Eradication rates of H. pylori infection decrease due to the emergence of resistance to antibiotics. Data on the antimicrobial susceptibility of H. pylori in Moroccan children are not available. This study aims to assess the prevalence of H. pylori infection and the metronidazole resistance rate of H. pylori in Moroccan pediatric patients, and their association with epidemiologic factors.

**Methods:**

a cross-sectional study was conducted on 132 pediatric patients who had an indication for upper gastrointestinal endoscopy and attended pediatric hospital Abderrahim Harouchi of the University Hospital Ibn Rochd, Casablanca, Morocco. Detection of H. pylori infection and the susceptibility to metronidazole was performed by classic PCR. Statistical analysis was performed using R Studio software.

**Results:**

the overall prevalence of H. pylori infection was 80.3%. vomiting was significantly associated with H. pylori infection (p-value=0.01). Regarding the resistance rate of metronidazole, we found that the prevalence of H. pylori resistance to metronidazole was high (70.8%) and it significantly increased, especially in pediatric patients living in urban areas (p-value=0.01).

**Conclusion:**

the prevalence of H. pylori infection and resistance rate of metronidazole were very high in Moroccan children. Therefore, triple therapy with metronidazole must be preceded by a study of the bacterium’s susceptibility to the prescribed antibiotics, in particular to metronidazole.

## Introduction

*Helicobacter pylori* (*H. pylori*) is a spiral, gram-negative, curved, microaerophilic bacterium. Infection with *H. pylori* is widespread throughout the world, usually occurring in childhood and remaining in the infected children for life unless appropriate treatment is applied [1,2]. It is the leading cause of chronic gastritis and peptic ulcer disease in children, and the prevalence of this infection ranges from 1.7% to 97.1% in children in different countries [3,4]. A study conducted in Morocco revealed that the prevalence of *H. pylori* infection in children was around 53% [5].

In Morocco, the first-line eradication therapy for *H. pylori* in children according to the European Society for Paediatric Gastroenterology Hepatology and Nutrition (ESPGHAN) and North American Society For Pediatric Gastroenterology, Hepatology and Nutrition (NASPGHAN) includes a proton pump inhibitor (PPI) and amoxicillin in combination with clarithromycin or metronidazole for two weeks [6]. Eradication success rates with this regimen have historically exceeded 90%. However, there has been a recent increase in the failure rate of this therapy due to the increasing resistance of *H. pylori* to antibiotics worldwide [7,8].

Metronidazole requires an activation step by reduction of the nitro group attached to the imidazole ring to achieve antimicrobial activity [9]. The reduction of this antibiotic is mainly mediated by oxygen-insensitive NADPH nitroreductase (*RdxA*), NADPH-flavin-oxidoreductase (*FrxA*), and ferredoxinlike enzymes (*FrxB*) in *H. pylori* [9]. The mechanism of resistance of *H. pylori* to metronidazole are complex and primarily due to the mutational inactivation of the *rdxA* and *FrxA* genes which encode the oxygen-insensitive NADPH nitroreductase and NADPH-flavin oxidoreductase respectively [10]. These mutations include frameshifts, deletions, premature truncations, and missense mutations within the two genes [10]. Inactivation of the *rdxA* by deletion of 200-bp in the *rdxA* encoding gene is the most common mechanism of metronidazole resistance [10,11].

Metronidazole resistance frequency in children varies depending on the geographic area and ranges from 10% to 91% [12]. In Morocco, a high prevalence of *H. pylori* resistance to metronidazole has been reported in adults (62.7%) [13]. To our knowledge, the prevalence of *H. pylori* resistance to metronidazole in Moroccan children has never been investigated by molecular test. Therefore, we conducted this study to assess the prevalence of *H. pylori* infection and the metronidazole resistance rate of *H. pylori* in Moroccan pediatric patients by targeting the *rdxA* gene deletion mutation in *H. pylori*, and their association with epidemiological factors.

## Methods

**Study design:** a cross-sectional study was conducted at Children´s Hospital Abderrahim Harouchi Casablanca, Morocco from September 2022 to October 2023. This study was carried out to evaluate the prevalence of *H. pylori* infection and the metronidazole resistance rate of *H. pylori* in Moroccan pediatric patients, and their association with epidemiologic factors.

**Study population and data collection:** the study involved 132 children (aged between 2 and 17 years old) referred for endoscopy for digestive symptoms such as nausea or vomiting, heartburn, epigastric pain, recurrent abdominal pain, digestive hemorrhage, weight loss, and refractory anemia in pediatric Hospital Abderrahim Harouchi of the University Hospital Ibn Rochd, Casablanca, Morocco. Patients younger than 2 years, use of antibiotics or PPI in the four weeks before endoscopy, and patients not born or living in Morocco were excluded from the present study. Upper endoscopy was conducted by experienced endoscopists and endoscopic findings were recorded during endoscopy. Four gastric biopsies were obtained from each patient, two from the antrum and two from the fundus, for molecular detection and histological examination. One of the four gastric biopsies was fixed in 10% formalin and embedded in paraffin, and histologic sections were then colored with hematoxylin-eosin and modified Giemsa. Histomorphologic evaluation was carried out following the updated Sydney System criteria [14] by the pathologist who determined the presence/absence of *H. pylori*, and gastric lesions (chronic gastritis, gastric atrophy, intestinal dysplasia, and intestinal metaplasia). Chronic inflammation intensity of inflammation was reported as none, mild, moderate, and severe. A questionnaire was used to collect information such as demographic characteristics of the participants, and history of family infection with *H. pylori*, and history of previous eradication of *H. pylori*. Informed consent was acquired from the patient's parents.

### Molecular test for *H. pylori* detection

**Deoxyribonucleic acid (DNA) extraction:** extraction of total DNA from gastric biopsies was performed using the isolate Genomic DNA Kit (Bioline, Memphis, USA) following the provided instructions, and the isolated DNA was stored at -20°C until use.

***H. pylori* infection:** the detection of *H. pylori* infection was performed by amplification of the ureC gene (296 bp) using specific primers (Table 1) [15]. Each reaction mixture was prepared in a final volume of 20 μL by adding dNTPs (0.5 mM), primers (0.5 μM of each primer), 300 ng of DNA, MgCl2 (1.5 mM), and 1 U Taq polymerase (My Taq DNA Polymerase, Bioline). The PCR thermal conditions were as follows: 95°C denaturation for 1 min, followed by 35 cycles at 95°C for 15 sec, 55°C for 30 sec, 72°C for 30 sec, and 72°C final elongation cycle for 7 min.

**Table 1:** primer sequences for detection of *H. pylori*

Genes	Forward primer	Reverse primer
ureC	AAGCTTTTAGGGGTGTTAGGGGTTT	AAGCTTACTTTCTAACACTAACGC
rdxA	AATTTGAGCATGGGGCAGA	GAAACCGCTTGAAAACCT

**Figure 1:**
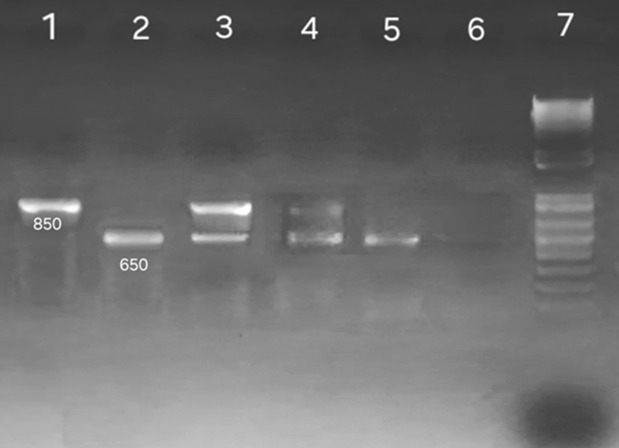
polymerase chain reaction products of the *rdxA* gene: lane 1 positive control, lane 2 resistant strain mutant gene 650bp, lanes 3 and 4 resistant strains with a wild and mutant gene, lane 6 negative control, lane 7 1Kb DNA ladder

**Metronidazole susceptibility detection:** resistance to metronidazole was determined by detecting the 200 bp deletion of the *rdxA* gene, which is one of the most common mechanisms of *H. pylori* metronidazole resistance [16]. Polymerase chain reaction (PCR) was carried out to amplify the *rdxA* gene using specific primers (Table 1). For susceptible strains (wild-type gene of *H. pylori*) PCR amplification of this gene results in the amplification of an 850 bp fragment. Conversely, for resistant strains (mutant gene of *H. pylori*), PCR results in the amplification of a 650 bp fragment. The presence of two genes type and mutant genes was considered resistance [16,17]. The PCR amplification of the *rdxA* gene was carried out in a final volume of 20 μL comprising 300 ng of DNA, primers (0.8 μM of each primer), dNTPs (0.5 mM), MgC2 (1.5 mM), and 1U of Taq polymerase (My Taq DNA Polymerase, Bioline). The PCR thermal condition consisted of pre-denaturation at 95°C for 1 min, followed by 35 cycles of 95°C for 15 sec, 58°C for 30 sec, 72°C for 30 sec, and finally an elongation at 72°C for 7 min. After that 5 μL of PCR products were added to 1 μL of loading buffer (10x) and electrophoresed on agarose gel 1.5% (Figure 1).

**Statistical analysis:** Rstudio for Windows version 4.3.1 was used for statistical analyses. Missing data of specific variables were excluded from the analysis, resulting in adjustments to the sample size for those variables. Descriptive data are expressed as frequencies and means ± standard deviations (SD). For continuous variables, differences between groups were assessed using the t-test or Kruskal test, and for categorical variables using the Chi-square or Fisher´s exact test. The differences were considered significant at p < 0.05.

**Ethical considerations:** the study was conducted according to the ethical standards of Helsinki and approved by the Ethics Committee of the Mohamed VI University of Health Sciences of Casablanca of Morocco (CE/UM6SS/18/24). Informed consent was obtained from the patient's parents.

## Results

**Patient characteristics:** this study included 132 biopsies obtained from children with a histopathological diagnosis of chronic gastritis, 65 were males (49.2%), and 67 were females (50.8%), with an overall mean age was 9.27 ± 3.4 years, ranging from 2 to 17 years. Children were stratified into two age groups: 66 children among the group of [2-9 years], and 66 among the group of [10-17 years]. Most of our patients lived in urban rather than rural areas (85.6% vs 14.4%, respectively). Nodular gastritis was the most frequent endoscopic finding (41.7%) in our study. Out of 132 patients included in this study, histologic examination results were available for 114 participants and revealed a predominance of chronic gastritis in 80.3%, while atrophic and follicular gastritis was found in 2.3% and 3.8% of patients respectively. The intensity of chronic inflammation showed that moderate gastritis was the predominant finding in 62.3% of patients, while mild gastritis and severe gastritis were found in 36.8% and 0.9% of patients respectively (Table 2).

**Table 2:** epidemiologic and clinical factors associated with *H. pylori* infection in children (n=132)

Characteristics	Total (%)	*H. pylori* positive (%)	*H. pylori* negative (%)	P-value
**Gender**				
Male	65 (49.2%)	53 (81.5%)	12 (18.5%)	0.72
Female	67 (50.8%)	53 (79.1%)	14 (20.9%)	
**Age (years)**				
2-9	66 (50%)	55 (83.3%)	11 (16.7%)	0.47
10-17	66 (50%)	51 (77.3%)	15 (22.7%)	
**The living area**				
Urban	113 (85.6%)	91 (80.5%)	22 (19.5%)	1
Rural	19 (14.4%)	15 (78.9%)	4 (21.1%)	
**History of previous eradication of *H. pylori***				
Yes	35 (26.5%)	27 (77.1%)	8 (22.9%)	0.49
No	97 (73.5%)	80 (82.5%)	17 (17.5%)	
**Family history of *H. pylori* infection**				
Yes	48 (36.4%)	39 (81.2%)	9 (18.8%)	0.96
No	84 (63.6%)	68 (81%)	16 (19%)	
**Endoscopy findings**				
Normal	35 (26.5%)	28 (80%)	7 (20%)	0.36
Nodular gastritis	55 (41.7%)	47 (85.5%)	8 (14.5%)	
Congestive gastritis	7 (5.3%)	4 (57.1%)	3 (42.9%)	
Petechial gastritis	33 (25%)	25 (75.8%)	8 (24,2%)	
Erythematous gastritis	2 (1.5%)	2 (100%)	0	
**Histological characteristics**				
N/A	18 (13.6%)	11 (61.1%)	7 (38.9%)	0.14
Chronic gastritis	106 (80.3%)	87 (82.1%)	19 (17.9%)	
Atrophic gastritis	3 (2.3%)	3 (100%)	0	
Follicular gastritis	5 (3.8%)	5 (100%)	0	
**Chronic inflammation**				
Mild	39 (36.8%)	29 (74.4%)	10 (25.6%)	0.33
Moderate	66 (62.3%)	57 (86.4%)	9 (13.6%)	
Severe	1 (0.9%)	1 (100%)	0	
**Symptoms**				
Vomiting	79 (59.8%)	69 (87.3%)	10 (12.7%)	0.01
Epigastric pain	92 (69.7%)	71 (77.2%)	21 (22.8%)	0.17
Recurrent abdominal pain	52 (39.4%)	44 (84.6%)	8 (15.4%)	0.31
Halitosis	72 (54.5%)	61 (84.7%)	11 (15.3%)	0.16
Digestive hemorrhage	26 (19.7%)	23 (88.5%)	3 (11.5%)	0.24
Refractory anemia	36 (27.3%)	32 (88.9%)	4 (11.1%)	0.12
Heartburn	10 (7.6%)	6 (60%)	4 (40%)	0.1

N/A: not available

**Prevalence of *H. pylori* infection and risk factors:** of the 132 patients included in this study, 106 (80.3%) were positive and 26 (19.7%) were negative for *H. pylori* infection based on PCR results for detection of the UreC gene. For the histological results among the 114 patients, 101 were positive for *H. pylori* infection (88.6%) and 13 (11.4%) were negative and it was not performed in 18 patients. Demographic data, history of previous eradication of *H. pylori*, family history of *H. pylori* infection, endoscopy findings, symptoms, and histological findings in *H. pylori* infected patients, compared to those who were uninfected, are presented in Table 2. The prevalence of *H. pylori* infection was higher in children aged between 2 and 9 years (83.3% VS 77.3%), but the difference was not statistically significant (p-value=0.47). Regarding the living area, *H. pylori* infection was higher in patients living in urban and rural areas (80.5%, and 78,9%, respectively) statistically, the difference was not significant. The distribution of *H. pylori* infection was roughly similar between females and males (79.1% vs 81.5% respectively). Vomiting as a presenting symptom was more common among infected children and the difference was statistically significant (p-value=0.01). There were no statistically significant differences between positive and negative children in terms of gender, and history of previous eradication of *H. pylori*, family history of *H. pylori* infection, endoscopy findings, and histological findings.

**Prevalence of metronidazole resistance:** several mechanisms were reported to contribute to *H. pylori* resistance to metronidazole in this study we investigate the frequency of *rdxA* gene deletion mutation in *H. pylori*. Our study revealed a high resistance rate to metronidazole of 70.8% (75/106) in infected patients, whereas only 29.2% (31/106) were susceptible to metronidazole.

**Resistance to metronidazole and risk factors:** the results of the study of risk factors contributing to metronidazole resistance are summarized in Table 3. The metronidazole resistance rate was higher in children older than 9 years (80.4% vs 61.8%) but this difference was not statistically significant (p-value=0.35). According to gender, the resistance rate in females was slightly higher (75.5 vs 66%). However, no significant difference was found between resistance to metronidazole and genders (p-value=0.28). In this study, we have also assessed the correlation between living areas and metronidazole resistance. Our data showed that children living in urban areas have the highest rate of metronidazole resistance (75.8%) compared to those from rural areas (40%) and the difference was statistically significant (p-value=0.01). The relationship between resistance and gastric lesions showed that chronic gastritis was higher in children infected with resistance strains (71.3%). However, the correlation between the severity of histological gastritis and metronidazole resistance did not reveal a statistically significant difference (p-value=0.35) (Table 3).

**Table 3:** epidemiological factors associated with *H. pylori* resistance to metronidazole in infected children (n=106)

Characteristics	Metronidazole resistance (%)	Metronidazole suceptibility (%)	P-value
**Gender**			0.28
Male	35 (66%)	18 (34%)	
Female	40 (75.5%)	13 (24.5%)	
**Age (years)**			0.35
2-9	34 (61.8%)	21 (38.2%)	
10-17	41 (80.4%)	10 (19.6%)	
**The living area**			0.01
Urban	69 (75.8%)	22 (24.2%)	
Rural	6 (40%)	9 (60%)	
**History of previous eradication of *H. pylori***			
Yes	22 (81.5%)	5 (18.5%)	0.15
No	53 (67.1%)	26 (32.9%)	
**Family history of *H. pylori* infection**			
Yes	26 (68. 4%)	12 (31.6%)	0.69
No	49 (72.1%)	19 (27.9%)	
**Histological characteristics**			
N/A	9 (81.8%)	2 (18.2%)	0.35
Chronic gastritis	62 (71.3%)	25 (28.7%)	
Atrophic gastritis	2 (66.7%)	1 (33.3%)	
Follicular gastritis	2 (40%)	3 (60%)	
**Chronic inflammation**			
Mild	21 (72.4%)	8 (27.6%)	1
Moderate	40 (70.2%)	17 (29.8%)	
Severe	1 (100%)	0	

N/A: not available

## Discussion

*H. pylori* infection represents a serious public health problem in Morocco, several studies reported prevalence is very high in children and adults [5,18,19]. Hence, it is very essential to know the incidences of its resistance to different antibiotics used for the treatment of this bacterium. In the present study, a high incidence of *H. pylori* infection (80.3%) was observed in the patients enrolled. Its rate was higher compared to our neighboring countries such as Tunisia [20], Egypt [21], and Nigeria [22], but lower than that reported in Vietnam [23]. There was a notable increase in the incidence of *H. pylori* infection in Moroccan children compared to previous studies, which reported rates of 45% [18] and 53% [5].

These differences in incidence could be probably attributed to the different methods of diagnosis, sample size, and characteristics of patients included in this study. The frequency of *H. pylori* infection was slightly higher in younger children aged between 2-9 years (83.3%) than in older children aged between 10-17 years (77.3%), which is consistent with the results of a study conducted by Polfan *et al*. that revealed a high frequency of *H. pylori* infection in children between 2 to 9 years old (54.5%) [24]. In Vietnam, *H. pylori* infection was higher in children under the age of 3 years old [25]. Meanwhile, other studies have reported an increase in the frequency of *H. pylori* infection with age in children [22,24]. Our findings could be explained by the high incidence of *H. pylori* infection in developing regions and the highest incidence occurs at younger ages, especially in the initial five years of life [26].

The prevalence of *H. pylori* infection is high in children living in urban areas (80.5%) similar to our results Ravelomanana *et al*. found that the infection rate is higher in children from urban areas (46.9%) than in rural areas (26.4%) [27]. The high rate could probably be due to municipal water networks as described in the study of Ravelomanana *et al*. [27] or to mothers' habits of transmitting bacteria to their children, such as the pre-mastication of food. *H. pylori* infection in pediatric patients may be clinically asymptomatic or associated with non-specific symptoms such as recurrent abdominal pain, epigastric pain, vomiting, and gastroesophageal reflux, which are common to a variety of childhood illnesses [28]. in this study, a significant association was found between *H. pylori* infection and vomiting (p-value=0.01). These results were consistent with those reported by Dore *et al*. where diarrhea and nausea/vomiting were significantly associated in children [29]. on the other hand, in Romania, they found that epigastric pain was the most common symptom in infected children [30]. To our knowledge, the antibiotic susceptibility profile of pediatric patients in Morocco has never been reported before. In the course of this study, we examined the metronidazole resistance rate of *H. pylori* in Moroccan pediatric patients and their association with epidemiologic factors.

Metronidazole is a frequently used first-line treatment to eradicate *H. pylori* infection. however, its efficacy is undermined by escalating resistance, significantly reducing its therapeutic value [31]. In this study, we found a high metronidazole resistance rate, affecting 70.8% of infected patients. Our result is almost similar to that found in China (68.4%) [32] but much higher than that documented in Vietnam (14.5%) [2], Iran (41.1%) [33], and Jordan (50%) [34], and lower than that found in Egypt (82%) [35]. The high incidence of metronidazole resistance may be attributed to its extensive utilization for parasitic infections in children [12]. A massive national screening is recommended to confirm the results of our study and to make a well-informed decision regarding the use of metronidazole in the treatment of *H. pylori* in Moroccan children. Analysis of the association of the demographic data and metronidazole resistance demonstrated that patients living in urban areas were at high risk of developing metronidazole-resistant, with a rate of 75.8% compared to patients living in rural areas (40%) (p-value=0.01). In contrast, Nguyen *et al*. found that metronidazole was significantly higher in rural than urban areas (respectively 74.3%, 57.9%, p-value=0.011) [36]. This difference could be attributed to the higher use of antibiotics in urban areas than in rural areas in our country given the availability and accessibility of antibiotics in urban areas. According to age, we found an upward trend in the resistance rate of metronidazole in older children (80.4%) than in younger children (61.8%) but statistically, the difference was not significant. Oleastro *et al*. reported in their study that older children aged 11-18 years had the highest rate of metronidazole resistance compared to the youngest children [37]. The possible correlation between the resistance to metronidazole and age could be explained by the increased prescription of this antibiotic for other infections with age for other infections [38].

This study has several limitations. First of all, this study was conducted in a single center and examined a limited number of children; therefore, further multicenter studies should be undertaken. Moreover, the antibiogram method is the reference method for antibiotic susceptibility testing, but we could not use this method because of its high risk of failure given the *H. pylori* specific growth requirements. In addition, there are other mechanisms of *H. pylori* resistance to metronidazole, such as mutations in the *rdxA* and *frxA* genes, which may also contribute to resistance to metronidazole and future studies will be conducted in this study population to investigate these mechanisms.

## Conclusion

In conclusion, our data showed a high incidence of *H. pylori* among Moroccan children. In addition, we have found that vomiting may be a symptom associated with *H. pylori* infection in children. Infected children also had an elevated rate of metronidazole resistance, which was higher in those living in urban areas. This study highlights the critical importance of the prevention and eradication of *H. pylori*. Nevertheless, Given the increasing rate of metronidazole resistance and the limited number of antibiotics available for *H. pylori* eradication in pediatric patients, it is beneficial to include antimicrobial susceptibility testing in the diagnostic protocol for *H. pylori* in our country.

### What is known about this topic

- *H. pylori infection is a chronic infection that affects more than half of the worldwide population;*
- *Metronidazole is one of the antibiotics used for the eradication of H. pylori in children*.

### What this study adds

- *The prevalence of H. pylori infection and resistance to metronidazole was high in Moroccan children;*
- *Children living in urban areas have the highest rate of H. pylori metronidazole resistance;*
- *Metronidazole should be prescribed based on susceptibility test results*.
